# Heat Stress and Anthropogenic Substrates: Molecular and Behavioral Adaptation of *Metridium senile* in Human-Modified Marine Environments

**DOI:** 10.3390/ijms26178415

**Published:** 2025-08-29

**Authors:** Guangliang Teng, Wen Chen, Xiujuan Shan, Qing Zhu, Xianshi Jin

**Affiliations:** 1 Key Laboratory of Sustainable Development of Marine Fisheries, Ministry of Agriculture and Rural Affairs, Yellow Sea Fisheries Research Institute, Chinese Academy of Fishery Sciences, Qingdao 266071, China; tenggl@ysfri.ac.cn (G.T.); chenwenbys@126.com (W.C.); zhuqing991105@163.com (Q.Z.); jin@ysfri.ac.cn (X.J.); 2 Shandong Changdao Fishery Resources National Field Observation and Research Station, Yantai 265800, China; 3 Laboratory for Marine Fisheries Science and Food Production Processes, Qingdao Marine Science and Technology Center, Qingdao 266237, China

**Keywords:** *Metridium senile*, heat stress, behavioral experiments, transcriptome analysis, adaptive mechanism

## Abstract

Marine litter provides novel habitats for substrate-dependent species, potentially facilitating their expansion under climate change. This study investigated the thermal adaptability and substrate selectivity of the cold-water sea anemone *Metridium senile* in the Yellow Sea, where rising temperatures and anthropogenic substrates may drive its proliferation. Behavioral experiments revealed diminished adhesion capacity under thermal stress (13 °C and 18 °C), with no substrate preference observed. Transcriptomic analysis identified 175 and 340 differentially expressed genes (DEGs) at 13 °C and 18 °C, respectively, compared with the control (8 °C). These DEGs were enriched in metabolic processes, oxidative stress, and cell homeostasis, with key pathways including dorso-ventral axis formation, ECM–receptor interaction, TGF-β, and Wnt signaling pathways. Notably, 7 regeneration-related, 20 adhesion-related, and 16 collagen-related DEGs were implicated in adaptive responses to heat stress. Our findings elucidate the molecular mechanisms underlying *M. senile*’s resilience and highlight its potential to exploit human-modified habitats under warming conditions, offering insights into ecological shifts in marine ecosystems.

## 1. Introduction

Anthropogenic activities have profoundly altered marine ecosystems through habitat modification, pollution, and climate-driven temperature shifts [1,2,3,4]. Rising sea temperatures and the proliferation of marine litter—plastic, metal, and discarded fishing gear—create novel substrates for benthic organisms, particularly sessile species such as sea anemones [2,5,6,7].

Sea anemones exhibit complex reproductive strategies and life history processes that are regulated by both genetic and environmental factors. Temperature is widely considered to influence the reproductive strategy and reproductive success of sea anemones, and extreme temperature changes can disrupt gamete production, fertilization and larval development, resulting in decreased reproductive success [8,9]. The cold-water sea anemone *Metridium senile*, which is widely distributed in temperate and subpolar regions, serves as a model for studying climate-driven ecological adaptations due to its ecological plasticity, rapid asexual reproduction, and adaptation to variable substrates [6,10,11]. In recent years, *M. senile* has not only shown a regional expansion trend in its native area but has also invaded the waters of the Southern Hemisphere [2,6,12,13]. Moreover, population expansion or biological invasion by *M. senile* may increase predation or competitive pressure on benthic animals, affecting the energy flow of benthic ecosystems [14].

The effective substrate and temperature are considered the limiting factors that restrict the dispersal of *M. senile* [2,10]. Previous studies have shown that many types of seafloor litter are significantly correlated with the distribution of *M. senile*, and it is speculated that these factors promote their regional expansion [6]. Glon et al. (2019) [15] conducted an environmental stress experiment to study the effects of temperature and salinity stress on the lethal temperature and reproductive status of *M. senile*. A previous study revealed that *M. senile* can survive at 18 °C and 20 °C, and under this heat stress, it exhibits asexual reproduction via pedal laceration; at relatively high temperatures, the mortality rate of *M. senile* significantly increases [15].

**Figure 1:**
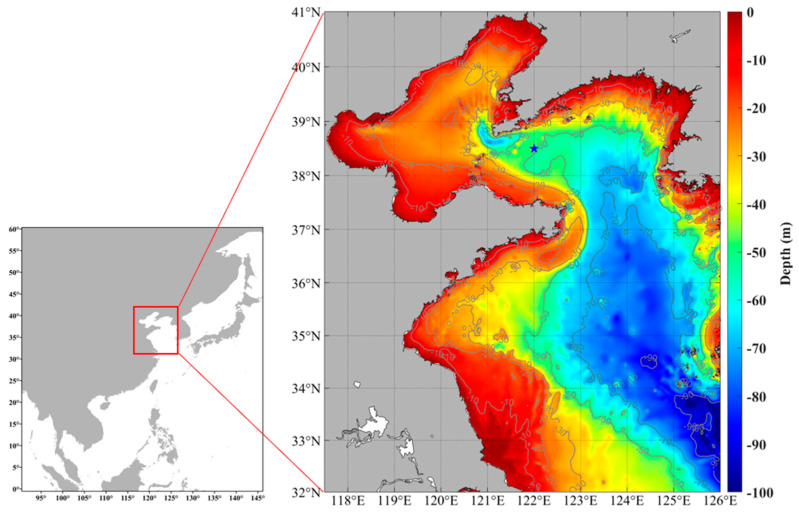
Sampling sites of *Metridium senile* in the Yellow Sea. Star symbols indicate collection localities; blue shading denotes the deep-water zone, which predominantly overlaps with the Yellow Sea Cold Water Mass (YSCWM).

The Yellow Sea, characterized by its unique hydrological environment, serves as the southern distribution boundary for *M. senile* and defines its thermal tolerance limit [16]. The Yellow Sea Cold Water Mass (YSCWM) is a low-temperature and high-salinity water body that lies in the Yellow Sea Trough during summer, covering an area of approximately 1.3 × 10^5^ km^2^ (Figure 1) [17]. From 1958 to 2016, the YSCWM exhibited significant interannual to decadal variations, characterized by a warming water trend and a decreasing volume [18]. Moreover, the frequency, intensity, and duration of marine heatwaves in the Yellow Sea have significantly increased in recent years [19]. Short-term extreme marine heatwaves can cause considerable thermal stress, disrupting marine ecosystem structure and function, particularly affecting the survival of cold-water species [3].

While field studies correlate substrate availability and temperature with *M. senile* distribution [3,6], the molecular and behavioral mechanisms enabling its persistence under thermal stress remain unresolved. The primary aim of this study was to elucidate the molecular and behavioral mechanisms underlying the persistence and potential expansion of the cold-water sea anemone *M. senile* under heat stress relevant to the warming YSCWM, with a specific focus on its interaction with anthropogenic substrates. To achieve this overarching aim, we address two critical and interconnected research objectives. 

Behavioral Response: We quantitatively assessed how elevated temperatures, simulating projected warming of the YSCWM (specifically 13 °C and 18 °C versus an 8 °C control), impact *M. senile*’s key ecological behaviors, namely, adhesion capacity and substrate selectivity, under controlled laboratory conditions. 

Molecular Response: We identified the transcriptional pathways and key gene expression changes associated with the adaptive response of *M. senile* to acute thermal stress (at 13 °C and 18 °C). We hypothesized that heat stress significantly alters the expression of genes involved in metabolism, reproduction, the stress response, and extracellular matrix (e.g., collagen) synthesis/degradation. By integrating behavioral assays with transcriptomic analysis, we sought to elucidate the mechanisms allowing *M. senile* to colonize anthropogenic substrates in warming seas. Our findings provide a framework for predicting species resilience in rapidly changing marine environments.

## 2. Results

### 2.1. Substrate Selectivity and Attachment Ability of M. senile

#### 2.1.1. Changes in the Attachment Ability of *M. senile*

**Figure 2:**
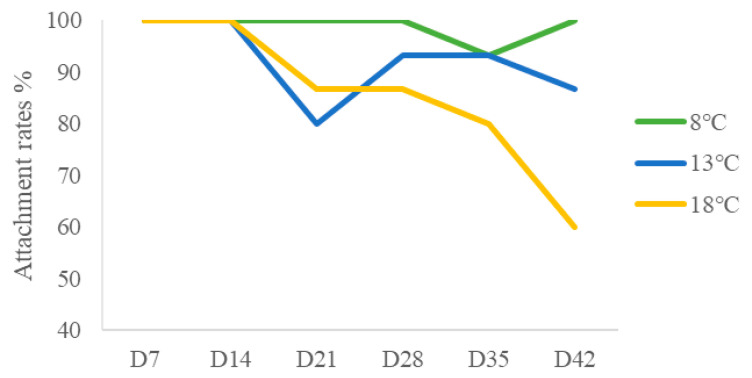
Attachment rates of *M. senile* under different heat stress conditions.

Throughout the experimental period, *M. senile* specimens in all groups maintained a 100% survival rate. Behavioral monitoring revealed that the attachment rate of *M. senile* in the control group remained stable throughout the experiment (Figure 2). During the behavioral experiments, the attachment rate at 18 °C was significantly lower than that at 8 °C and 13 °C (*p* < 0.05), whereas there was no significant difference between 8 °C and 13 °C (*p* > 0.05). As the duration of heat stress increased, the attachment rate of *M. senile* in the experimental groups tended to decrease, with the 18 °C group showing a more pronounced decline compared to the 13 °C group (Figure 2).

#### 2.1.2. Selectivity of *M. senile* for Different Substrate Materials

**Figure 3:**
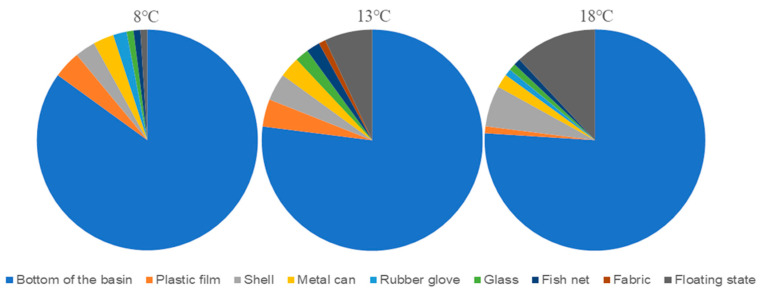
Selectivity of *M. senile* for different substrate types.

Analysis of the selectivity of different groups of *M. senile* for different substrates throughout the experimental period (Figure 3) revealed that the *M. senile* in the 8 °C group was primarily attached to the bottom of the basin, with an attachment rate of 86%. This was followed by plastic film (with an attachment rate of 4%), shell and metal can (with an attachment rate of 3%), rubber gloves, glass, fishing nets, and no substrate (floating state). In the 13 °C group, 7% of the *M. senile* floated, and 78% attached to the bottom of the basin. This was followed by plastic film and shells (with an attachment rate of 4%), metal cans (with an attachment rate of 3%), glass, fishing nets, and fabric. In the 18 °C group, 12% of the *M. senile* floated, and 76% of the *M. senile* attached to the bottom of the basin. This was followed by shells (with an attachment rate of 6%), metal cans, plastic films, glass, fishing nets, and rubber gloves. In addition, behavioral observation experiments indicated that *M. senile* had poor mobility, with 80% being attached to the bottom of the basin. All the experimental groups of *M. senile* showed a lack of selectivity toward different substrates.

### 2.2. Transcriptomic Analysis Results

#### 2.2.1. Transcriptome Data Quality and Gene Statistics

The comprehensive analysis of various detection indicators revealed that the RNA quality of nine samples was Grade A, indicating that the RNA quality was suitable for sequencing. After low-quality raw reads were excluded, a total of 61.27 Gb of clean reads were obtained. After the transcriptome sequence of each sample was filtered, a total of 38.37~53.15 × 10^6^ effective reads were obtained, with an effective read data range of 5.71~7.94 Gb and an effective read proportion exceeding 99.12%. The proportion of Q30 exceeded 93.52%, and the GC content accounted for 41.51~41.94% of the total bases (Table S2). After Trinity (https://github.com/trinityrnaseq/trinityrnaseq/releases, accessed on 3 July 2025) splicing and assembly, a total of 67,207 unigenes were obtained from the *M. senile* transcriptome. The length range of the unigenes was 201–46,469 bp, with an average length of 957 bp and an N50 of 1712 bp (Figure S1).

We aligned and annotated 67,207 nonredundant unigenes with known gene sequences in four databases (Nr, KEGG, COG/KOG, and SwissProt). Specifically, 26,579 unigenes were annotated in the Nr database, 26,075 unigenes in the KEGG database, 11,165 unigenes in the COG/KOG database, and 13,158 unigenes in the SwissProt database (Figure S2).

#### 2.2.2. DEG Analysis and Annotation

**Figure 4:**
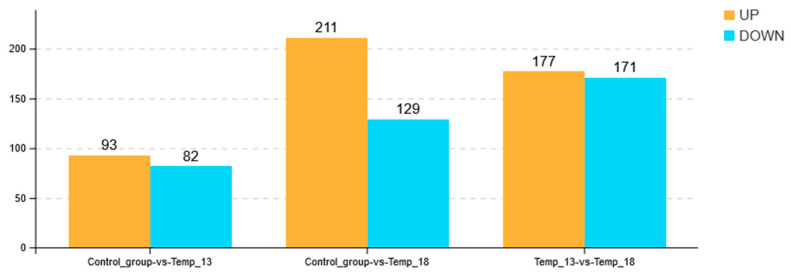
Histogram of unigene expression in different groups.

After the overall expression levels of the unigene sequences across all the experimental groups were normalized, the expression levels of the unigene sequences in the 8 °C experimental group were used as a control. By setting two parameters, |log_2_FC| > 2 and FDR < 0.05, significantly differentially expressed unigene sequences were subsequently screened from other temperature gradient experimental groups (13 °C, 18 °C). The results revealed significant differences in the expression levels of 175 (93 upregulated and 82 downregulated) and 340 (211 upregulated and 129 downregulated) unigene sequences in the 13 °C and 18 °C experimental groups, respectively (Figure 4).

**Figure 5:**
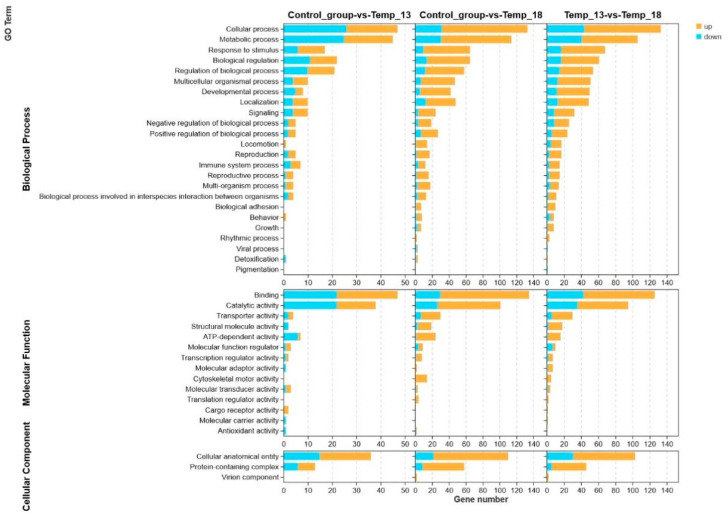
Secondary histogram of the GO enrichment classification results for the DEGs.

GO enrichment analysis revealed that the DEGs were annotated to 41 subcategories across three major categories: biological process (BP), molecular function (MF), and cell component (CC) (Figure 5). Among these, most DEGs were annotated to the cellular process, metabolic process, response to stimulus, and biological regulation terms in the biological process category. In the molecular function category, most DEGs were annotated to binding, catalytic activity, and transporter activity. In the cell component category, most DEGs were annotated to cellular anatomical entities and protein-containing complexes.

**Figure 6:**
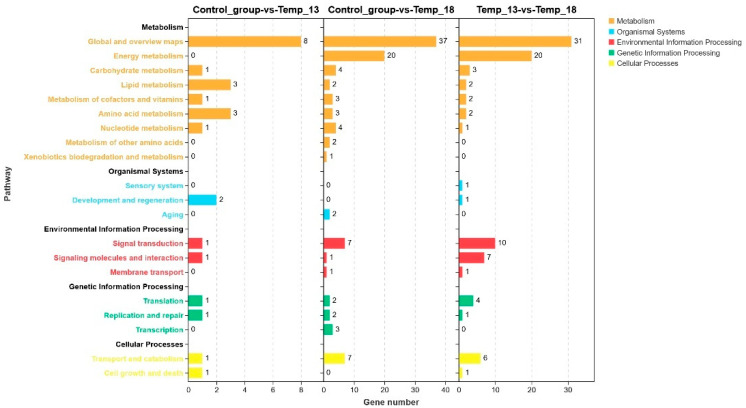
Bar chart showing the enriched KEGG pathways of the DEGs.

The KEGG pathway enrichment analysis revealed that the DEGs were annotated to a total of 60 pathways across 5 major categories and 20 subcategories (Figure 6). KEGG pathway enrichment analysis revealed distinct distribution patterns of DEGs across functional hierarchies. Among the five major categories, Metabolism contained the highest number of annotated DEGs, with dominant enrichment in four subcategories: (1) Global and overview maps, (2) Energy metabolism, (3) Carbohydrate metabolism, and (4) Lipid metabolism. Within the Organismal Systems category, Development and regeneration emerged as the most prominently enriched subcategory. For Environmental Information Processing, significant enrichment occurred in Signal transduction and Signaling molecules and interactions. The Cellular Processes category showed primary enrichment in Transport and catabolism and Cell growth and death subcategories.

**Figure 7:**
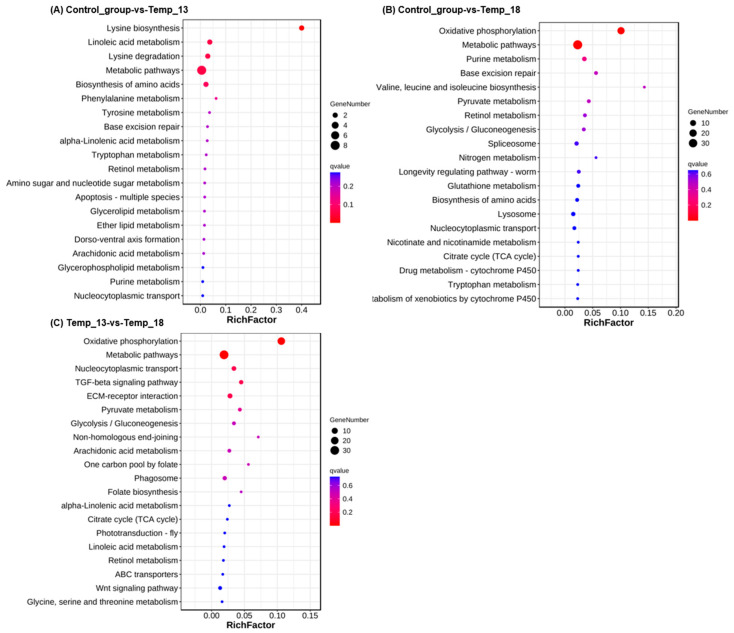
Top 20 KEGG-enriched pathways of DEGs between groups. Note: (**A**) control group vs. 13 °C group; (**B**) control group vs. 18 °C group; (**C**) 13 °C group vs. 18 °C group.

KEGG pathway analysis revealed significant enrichment of differentially expressed genes (DEGs) in the following comparisons: Control vs. Temp_13 DEGs were enriched in metabolic pathways, lysine biosynthesis, linoleic acid metabolism, lysine degradation, and biosynthesis of amino acids (Figure 7); Control vs. Temp_18 DEGs showed enrichment in metabolic pathways and oxidative phosphorylation; Temp_13 vs. Temp_18 DEGs demonstrated enrichment in metabolic pathways, oxidative phosphorylation, nucleocytoplasmic transport, TGF-beta signaling pathway, and ECM–receptor interaction.

#### 2.2.3. Screening of DEGs Related to the Adaptive Response

To identify the potential functional genes and signaling pathways involved in temperature regulation, we analyzed the differentially expressed gene set by searching for keywords related to “regeneration,” “adhesion,” and “collagen and fibrinogen”. The results revealed that a total of 7 significant DEGs were associated with regenerative function (Table S3), 20 significantly DEGs were related to adhesion function (Table S4), and 16 significantly DEGs were linked to collagen function (Table S5). Furthermore, DEGs related to adaptive response were significantly enriched in key signaling pathways, including dorso-ventral axis formation, ECM–receptor interaction, TGF-beta, and Wnt signaling (Table S6).

**Figure 8:**
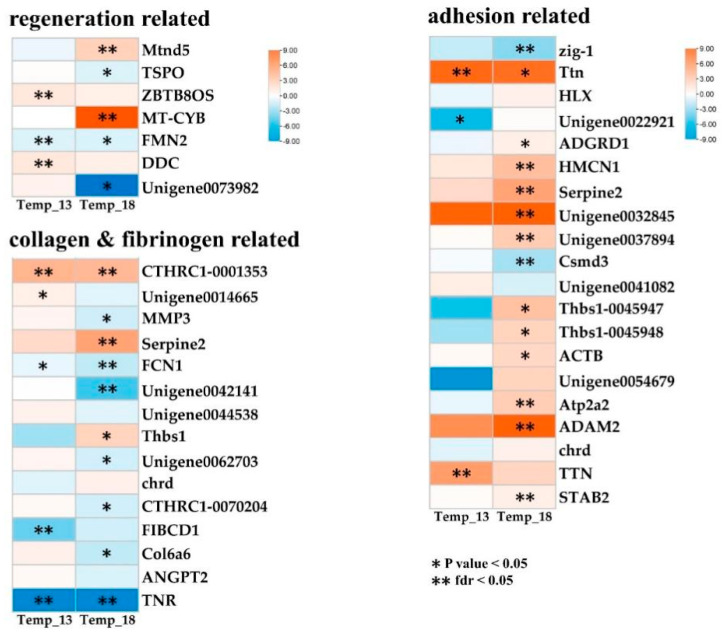
DEG expression response patterns. * indicates|log_2_FC| > 2 and *p* < 0.05; ** indicates |log_2_FC| > 2 and *p* value and FDR < 0.05.

To further explore the molecular mechanisms underlying the adaptive regulation of the growth, reproduction, and adhesion capabilities of *M. senile* under heat stress conditions, an expression pattern analysis was conducted on DEGs corresponding to the keywords “regeneration”, “adhesion”, and “collagen and fibrinogen” (Figure 8). First, among the genes related to “regeneration”, four genes were significantly upregulated, and three genes were significantly downregulated. Specifically, *Mtnd5* and *MT-CYB* exhibited upregulation, while *FMN2* and *TSPO* were downregulated. Among the genes related to “adhesion”, 13 genes showed significant upregulation versus 3 downregulated, with key upregulated genes including *Ttn*, *HMCN1*, *Serpine2*, *Unigene003789*, *ADAM2*, and *TTN*. Downregulated genes comprised *zig-1* and *Csmd3*. Among the genes related to “collagen and fibrinogen”, four were upregulated and eight downregulated. Upregulated genes included *CTHRC1-0001353* and *Serpine2*, while downregulated genes featured *MMP3*, *FCN1*, *FIBCD1*, and *TNR*. Furthermore, a review of the overall expression patterns of the DEGs revealed that the maximum number of significant DEGs occurred at 18 °C, indicating that genes related to *M. senile* growth, reproduction, and adhesion capabilities presented a more positive response as the temperature increased.

#### 2.2.4. Validation of the RNA-Seq Results Using RT-PCR Methods

**Figure 9:**
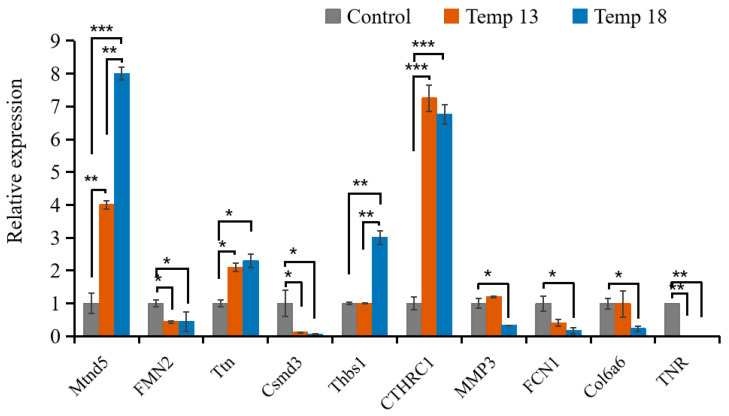
Comparisons between RNA-Seq data and RT-PCR results. *, ** and *** indicate *p* < 0.05, *p* < 0.01 and *p* < 0.001, respectively.

This figure compares expression patterns of ten target DEGs across three temperature treatments (Control, 13 °C, 18 °C) (Figure 9). The expression patterns were consistent with those obtained from RNA-Seq (*p* < 0.05) between different temperature treatments, confirming the accuracy and reliability of the transcriptome sequencing results (Figure 8 and Figure 9).

## 3. Discussion

The cold-water sea anemone *M. senile* faces increasing threats from climate-driven warming and habitat modification. Here, we integrated behavioral and transcriptomic analyses to elucidate adaptive mechanisms under heat stress, revealing the molecular and functional trade-offs that may underpin its persistence in human-altered environments. In the following discussion, we situate these findings within broader ecological and molecular contexts.

### 3.1. Thermal Stress Disrupts Adhesion Through Collagen Degradation

Behavioral observations revealed that approximately 80% of *M. senile* remained attached to or near their initial placement area (bottom of the basin). Given the limited substrate availability within the experimental setup relative to the full basin area, coupled with constrained movement capacity, all experimental groups presented minimal substrate selectivity. Critically, the lack of substrate selectivity of *M. senile* under heat stress contrasts with its documented preference for hard substrates in stable environments [2,20]. This behavioral plasticity aligns with its classification as a hemisessile organism capable of detachment and reattachment in response to environmental fluctuations, including hypoxic events and predator/prey encounters [21,22]. Such adaptive flexibility likely facilitates colonization of diverse anthropogenic substrates prevalent in climate-impacted coastal zones, including marine litter, thereby influencing population dynamics of attached organisms. Notably, the species’ ability to utilize pervasive marine debris (plastic litter, fishing gear, metallic structures) may accelerate its geographical expansion, mirroring invasion patterns observed in similar substrate-generalist ascidians and bryozoans [6,12,13,23].

Our experiments demonstrated that adhesion capacity decreases significantly at 18 °C, which coincides with the downregulation of collagen-related genes (*COL6A6*, *TNR*) and extracellular matrix proteases (*MMP3*) [24]. Similar patterns are observed in heat-stressed corals, where collagen degradation compromises structural integrity [25]. Notably, the upregulation of *CTHRC1*—a gene implicated in fibrosis and tissue repair [26]—suggests a compensatory mechanism to mitigate thermal damage. These findings parallel studies on *Aiptasia pulchella*, where heat stress disrupts adhesion through cytoskeletal reorganization [27]. Additionally, the concurrent downregulation of titin (*TTN*) and thrombospondin (*THBS1*), which are critical for maintaining muscle and extracellular matrix stability [28,29], further underscores the vulnerability of adhesion-related structures under thermal stress. Furthermore, this study identified significant enrichment of the DEGs THBS1, CTHRC1, and COL6A6 in the ECM–receptor interaction pathway, thereby underscoring the functional significance of this pathway and the pivotal roles of these key genes in the adhesion biology of *M. senile*. Such molecular shifts may explain *M. senile*’s increased detachment rates, a survival strategy to avoid heat stress, albeit at the cost of reduced habitat fidelity. Overall, however, despite temperature limitations, *M. senile* may become a potential “winner” in the human-modified marine environment due to its lack of substrate selectivity and transcriptional adaptability.

### 3.2. Metabolic and Reproductive Trade-Offs Under Heat Stress

Transcriptomic data revealed that heat stress reprograms *M. senile*’s metabolism, increasing the number of pathways linked to amino acid and lipid catabolism. This finding aligns with observations in other marine invertebrates, where elevated temperatures increase energy demands for cellular homeostasis [30]. The significant induction of cytochrome P450 (*CYPs*) genes highlights enhanced detoxification and antioxidant activity, likely countering reactive oxygen species (ROS) accumulation—a common consequence of metabolic hyperactivity [31,32]. However, prolonged upregulation of these pathways may deplete energy reserves, impairing long-term fitness.

Under heat stress, *M. senile* exhibits a complex trade-off between metabolic processes and reproductive activities. While mitochondrial genes (*mtnd5*, *mt-cyb*) involved in oxidative phosphorylation were upregulated—potentially supporting rapid fission—the downregulation of *FMN2*, a regulator of actin nucleation and DNA repair [33,34], suggests compromised cellular integrity during fission. This study revealed that under heat stress, the dorso-ventral axis formation pathway exhibited significant downregulation of FMN2. As a formin family protein, FMN2 mediates directional migration of basal disc cells to initiate bud formation and ultimately generate functional regenerated individuals. The marked downregulation of FMN2 may thus contribute to the observed suppression of asexual reproduction in *M. senile* during this experiment. In addition, KEGG pathway enrichment analysis of DEGs in *M. senile* showed significant differences in the expression levels of TGF-β, Wnt, and FoxO. The Wnt signaling pathway has been shown to have extensive relevance to tissue regeneration and asexual reproduction in cnidarians [35,36]. Components of the TGF-β are differentially expressed during the division process of *Nematostella vectensis*, indicating that they are important regulators of transverse fission [37]. Both the TGF-β and Wnt pathways are crucial for regulating epithelial-mesenchymal transition. The Wnt/β-catenin pathway regulates the pluripotent differentiation of stem cells, organ development, and regeneration, functionally resembling the Hippo, Notch, and TGF-β pathways [38]. In addition, FoxO mediates oxidative stress resistance and longevity, whereas Hippo controls cell proliferation and organ size [35,38]. Their coregulation suggests a mechanism to limit tissue damage while maintaining regenerative capacity. In summary, enrichment of these pathways also underscores their role in balancing the stress response and reproductive strategy, a mechanism conserved across cnidarians.

## 4. Materials and Methods

### 4.1. Sample Collection and Experimental Design

All *M. senile* samples were collected via fishery resource bottom trawl surveys at 38.5° N, 122° E during a November 2022 Yellow Sea survey, using a trawling speed of 3 knots/hour for a duration of 1 h (Figure 1). The overall experimental process was divided into the following stages: (1) a gradual gradient cooling acclimation phase in the early stage; (2) behavioral monitoring experiments conducted after the temperature stabilized at the predetermined level; and (3) transcriptomic experiments of *M. senile* after the behavioral monitoring experiments.

The aquarium consisted of a circular plastic basin with a diameter of 94 cm and a height of 31 cm. A total of 3 groups of sea anemones were set up, with 15 *M. senile* individuals randomly placed in each group. During the experiment, *M. senile* was fed fresh shrimp and shellfish regularly, and food residues were promptly disposed of to ensure that the water environment was clean. Temperature control during the aquaculture process was achieved through a temperature controller (HAILEA HC-100A, Guangdong, China) that could perform heating and cooling. Behavioral monitoring is conducted through a 4k high-definition camera (QIAODU, Fujian, China) for real-time monitoring.

#### 4.1.1. Temperature Acclimation Before the Behavioral Experiments

To prevent stress or mortality caused by rapid cooling in *M. senile*, the experimental groups were subjected to gradual temperature acclimation before the behavioral experiments began. The seawater temperature was decreased at a rate of 2 °C per day until it reached the target temperatures for each experimental group: 8 °C (control group), 13 °C, and 18 °C (experimental groups).

#### 4.1.2. Behavioral Analysis of the Substrate Selectivity and Attachment Ability of *M. senile*

**Figure 10:**
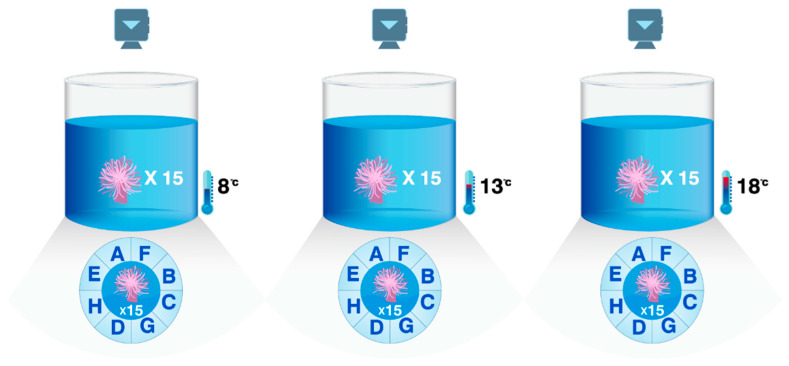
Experimental diagram of the selectivity and attachment of *M. senile* to different substrate types (Note: A: plastic film, B: metal can, C: glass, D: fish nets, E: metal can, F: rubber glove, G: fabric, and H: shell).

Behavioral experiments on the substrate selectivity and attachment ability of *M. senile* were conducted after the temperature acclimation phase. Different substrate types with the same surface area were used: A: plastic film, B: metal can, C: glass, D: fish nets, E: metal can, F: rubber glove, G: fabric, and H: shell (Figure 10). These types of substrates were randomly arranged with uniform spacing. The behavioral experiments were conducted with a complete cycle of 7 days. The attachment rate among the different temperature groups was used as an indicator of the attachment ability of *M. senile*. The main steps of each cycle included detaching the *M. senile*, releasing them in the center of the aquarium, and observing their attachment, movement, and individual conditions (floating or attached) through a camera. On the 7th day, the attachment rates of the *M. senile* in each group and their selective attachment to different substrates were statistically analyzed.

The selective substrate attachment experiment was conducted six times, and the net behavioral monitoring time was 42 days. Notably, although the behavioral monitoring experiment did not set up replicates, the six cycles of experimental observations can be regarded to a certain extent as replicates under different temperature conditions. To compare the effects of heat stress on the attachment of *M. senile*, this study used a generalized linear model (GLM) for binomial regression analysis, with the number of attached individuals in each cycle as the response variable and temperature as the fixed effect. The significance of the temperature effect was assessed via a likelihood ratio test, and post-hoc multiple comparisons between groups were adjusted using Tukey’s method.

### 4.2. Transcriptomic Experiments and Analysis

To avoid the potential effects of detachment and measurement on *M. senile* during the behavioral experiments and to further investigate the mechanisms by which heat stress influences adaptive behavior and adaptability in *M. senile*, heat stress was continuously maintained after the behavioral observation experiments were completed, the heat stress was continuously maintained. After a continuous heat stress period of 3 months, whole-tissue samples of *M. senile* were collected for transcriptomic analysis. To avoid analytical errors caused by individual differences, three biological replicates were taken from each group for transcriptome analysis. The detailed experimental procedures are described below.

#### 4.2.1. Acquisition of Sequencing Samples for Whole-Tissue Transcriptome Analysis

*M. senile* was placed on ice for anesthetization by freezing, and sterilized scissors and tweezers were used to quickly obtain tissues such as the foot disk, tentacles, septum, and gonads, as well as the intestinal contents. After rapid freezing in liquid nitrogen, the samples were stored in a −80 °C ultralow temperature freezer for subsequent RNA extraction experiments. To prevent cross-contamination between samples, dissection tools were thoroughly cleaned and disinfected with anhydrous ethanol prior to processing each sample. All the samples were disposed of in a safe and ethical manner following the completion of the experiments, according to standard laboratory protocols.

#### 4.2.2. RNA Extraction and Quality Control

Before RNA extraction, the foot disk, tentacles, septum, gonads, and intestinal contents of the *M. senile* were ground with liquid nitrogen and mixed in equal amounts to form a mixed sample. A TRIzol Reagent Kit (Huayueyang Biotech Co., Ltd., Beijing, China) was used according to the manufacturer’s instructions to extract total RNA from the mixed sample. Agarose gel electrophoresis and a Nanodrop microspectrophotometer were used to detect and analyze the RNA concentration and integrity of the sample for transcriptome sequencing.

#### 4.2.3. cDNA Library Construction and Transcriptome Sequencing

*M. senile* transcript library construction, sequencing, and assembly were performed by Gene Denovo Bioinformatics Technology Co., Ltd. (Beijing, China). The reagent kit used for library construction was the NEBNext^®^ UltraTM RNA Library Prep Kit from Illumina (San Diego, CA, USA), using mRNA as a template to construct double-stranded cDNA. After library inspection, sequencing was performed via an Illumina HiSeq instrument.

#### 4.2.4. Splicing and Annotation of Transcripts

To ensure the reliability of the analysis, the obtained raw data (raw reads) were filtered to remove reads containing adapters, poly-N sequences (where N represents undetermined base information), and low-quality reads (reads with Qphred ≤20, where the number of N bases accounted for more than 50% of the entire read length); this resulted in high-quality clean data for subsequent analysis. Trinity was used to assemble the clean data, Corset (https://github.com/Oshlack/Corset, accessed on 3 July 2025) was used to aggregate redundant transcripts, and BUSCO (https://busco.ezlab.org/, accessed on 3 July 2025) was used to perform quality assessment on the assembled Trinity.fasta, unigene.fa, and cluster.fasta files.

The nonredundant transcripts were annotated based on the Nr, Nt, Pfam, KOG, Swiss-Prot, KO, and GO databases, with the confidence ranking as follows: Nr > GO > KO > Swiss-Prot > Pfam > KOG > Nt.

#### 4.2.5. Gene Annotation and Gene Enrichment Analysis

To identify transcriptomic differences in *M. senile* after high heat stress, RSEM (https://deweylab.github.io/RSEM/README.html, accessed on 3 July 2025) was used to calculate gene expression counts for each sample, and the FPKM method was employed to calculate gene expression levels, with correction for sequencing depth. DEGseq2 was used to screen for differentially expressed genes (DEGs), with screening criteria of |log_2_(FC)| > 2 and FDR < 0.05.

In this study, Blast2GO software (https://www.blast2go.com/, accessed on 4 July 2025) was used with the Nr and Swiss-Prot protein databases to identify protein sequences with the highest sequence similarity to DEG sequences. Functional enrichment analysis and pathway enrichment analysis were conducted on the DEGs in each experimental temperature group based on the GO and KEGG databases, respectively. R software (https://www.r-project.org/, accessed on 4 July 2025) was used to visualize the results of functional enrichment and pathway enrichment analyses involving DEGs under different heat stress conditions.

### 4.3. Data Validation by Quantitative Real-Time PCR (RT-PCR)

Ten DEGs were selected post-sequencing. During the verification process, the 18S rRNA gene was used as the housekeeping gene, and gene expression levels were analyzed using the 2^−ΔΔCt^ method. The relevant primers were designed using Primer Premier 7 and synthesized and purified by Shanghai Sangon Biotech Co., Ltd. (Shanghai, China) (Table S1). The One Step TB Green PrimeScriptTM RT-PCR Kit II reagent kit (TaKaRa, Kyoto, Japan) was selected to prepare the reaction system for the RT-PCR reaction. Three replicates were performed for each sample. The gene expression differences were analyzed using the one-way ANOVA method, and the significance of these differences was examined using the *t*-test.

### 4.4. Ethical Statement

This study was conducted in accordance with animal ethical considerations and approved by the Institutional Animal Care and Use Ethics Committee of the Yellow Sea Fisheries Research Institute, Chinese Academy of Fishery Sciences (YSFRI-2022011).

## 5. Conclusions

The adaptive behavior and mechanism of *M. senile* under heat stress were studied via behavioral and transcriptomic experiments. In terms of behavioral responses, *M. senile* did not exhibit any selective preferences for different substrate types throughout the entire experimental period. Heat stress was found to directly impair the adhesion ability of *M. senile*, with this effect becoming progressively more pronounced over time. This study investigated the molecular mechanisms underlying the impact of heat stress on the biological functions of *M. senile*. A series of DEGs with significant changes in expression associated with asexual reproduction and adhesion ability were identified. These findings not only enhance our understanding of the adaptive response mechanisms in sea anemones and potentially other coelenterates but also hold significant implications for forecasting the expansion of widespread species amidst global changes.

## Data Availability

All the raw transcriptome data have been deposited into the NCBI Sequence Read Archive under Accession ID SUB15024102 (https://www.ncbi.nlm.nih.gov (accessed on 8 May 2025)).

## Supplementary Materials

The following supporting information can be downloaded at: https://www.mdpi.com/article/10.3390/ijms26178415/s1.

## References

[1] Jurgens L. (2018). Poleward range expansion of a non-indigenous bryozoan and new occurrences of exotic ascidians in southeast Alaska. BioInvasions Rec..

[2] Glon H., Daly M., Carlton J.T., Flenniken M.M., Currimjee Z. (2020). Mediators of invasions in the sea: Life history strategies and dispersal vectors facilitating global sea anemone introductions. Biol. Invasions.

[3] Gomes D.G.E., Ruzicka J.J., Crozier L.G., Huff D.D., Brodeur R.D., Stewart J.D. (2024). Marine heatwaves disrupt ecosystem structure and function via altered food webs and energy flux. Nat. Commun..

[4] Ibrahim I.A., Rawindran H., Alam M.M., Leong W.H., Sahrin N.T., Ng H.-S., Chan Y.J., Abdelfattah E.A., Lim J.W., Aliyu U.S. (2024). Mitigating persistent organic pollutants from marine plastics through enhanced recycling: A review. Environ. Res..

[5] Richardson A.J., Bakun A., Hays G.C., Gibbons M.J. (2009). The jellyfish joyride: Causes, consequences and management responses to a more gelatinous future. Trends Ecol. Evol..

[6] Teng G., Jin X., Fu C., Guan L., Jin Y., Chen Y., Yang T., Ding Q., Shan X. (2021). Is seafloor litter contributing to sea anemone blooms?. Sci. Total Environ..

[7] Ramalhosa P., Monteiro J.G., Rech S., Gestoso I., Álvarez S., Gizzi F., Parretti P., Castro N., Almeida S., Jiménez J.L. (2025). The role of marine debris as a vector, dispersal agent, and substrate for non-indigenous species on Oceanic Islands (Northeast Atlantic). Mar. Pollut. Bull..

[8] Ryan W.H. (2018). Temperature-dependent growth and fission rate plasticity drive seasonal and geographic changes in body size in a clonal sea anemone. Am. Nat..

[9] Ryan W.H., Adams L., Bonthond G., Mieszkowska N., Pack K.E., Krueger-Hadfield S.A. (2019). Environmental regulation of individual body size contributes to geographic variation in clonal life cycle expression. Mar. Biol..

[10] Bucklin A. (1987). Growth and asexual reproduction of the sea anemone *Metridium*: Comparative laboratory studies of three species. J. Exp. Mar. Biol. Ecol..

[11] Martin J.P., Garese A., Sar A.M., Acuña F.H. (2015). Fouling community dominated by *Metridium senile* (Cnidaria, Anthozoa, Actiniaria) in Bahía San Julián (Southern Patagonia, Argentina). Sci. Mar..

[12] Gimenez L.H., Battini N., González-Muñoz R., Glon H. (2023). Invader in disguise for decades: The plumose sea anemone *Metridium senile* in the Southwestern Atlantic Ocean. Biol. Invasions.

[13] Molinet C., Häussermann V., Astorga M., Barahona N., Espinoza K., Diaz M., Díaz P., Henríquez J., Matamala T., Soto D. (2023). Population expansion of the invasive sea anemone *Metridium senile* in the spatial mesoscale of a sea urchin bed in north-western Patagonia. Biol. Invasions.

[14] Teng G., Shan X., Jin X. (2023). Cascade effects of seafloor litter on benthic ecosystems in the northern Yellow Sea. Front. Mar. Sci..

[15] Glon H., Haruka Y., Daly M., Nakaoka M. (2019). Temperature and salinity survival limits of the fluffy sea anemone, *Metridium senile* (L.) in Japan. Hydrobiologia.

[16] Li Y., Xu K.D. (2020). Species diversity and faunal characteristics of the order Actiniaria (Cnidaria: Anthozoa) in the seas of China. Oceanol. Limnol. Sin..

[17] Su J.L., Huang D.J. (1995). On the current field associated with the yellow sea cold water mass. Oceanol. Limnol. Sin. Suppl..

[18] Li H., Zhai F.G., Dong Y.J., Liu Z., Gu Y., Bai P. (2024). Interannual-decadal variations in the Yellow Sea Cold Water Mass in summer during 1958—2016 using an eddy-resolving hindcast simulation based on OFES2. Cont. Shelf Res..

[19] Li Y., Ren G.Y., Wang Q.Y., Mu L., Niu Q., Su H. (2023). Record-breaking marine heatwave in northern Yellow Sea during summer 2018: Characteristics, drivers and ecological impact. Sci. Total Environ..

[20] Anthony K.R.N., Svane I. (1995). Effects of substratum instability on locomotion and pedal laceration in *Metridium senile* (Anthozoa: Actiniaria). Mar. Ecol. Prog. Ser..

[21] Wahl M. (1985). *Metridium senile*: Dispersion and small scale colonization by the combined strategy of locomotion and asexual reproduction (laceration). Mar. Ecol. Prog. Ser..

[22] Wahl M. (1985). The recolonization potential of *Metridium senile* in an area previously depopulated by oxygen deficiency. Oecologia.

[23] Pinochet J., Urbina M.A., Lagos M.E. (2020). Marine invertebrate larvae love plastics: Habitat selection and settlement on artificial substrates. Environ. Pollut..

[24] Pesheva P., Probstmeier R. (2000). The yin and yang of tenascin-R in CNS development and pathology. Prog. Neurobiol..

[25] Pinzón J.H., Kamel B., Burge C.A., Harvell C.D., Medina M., Weil E., Mydlarz L.D. (2015). Whole transcriptome analysis reveals changes in expression of immune-related genes during and after bleaching in a reef-building coral. R. Soc. Open Sci..

[26] Cao M., Ke D., Zhou H. (2024). The role and molecular mechanism of CTHRC1 in fibrosis. Life Sci..

[27] Sawyer S.J., Muscatine L. (2001). Cellular mechanisms underlying temperature-induced bleaching in the tropical sea anemone *Aiptasia pulchella*. J. Exp. Biol..

[28] Misaka T., Yoshihisa A., Takeishi Y. (2019). Titin in muscular dystrophy and cardiomyopathy: Urinary titin as a novel marker. Clin. Chim. Acta.

[29] Jiang D., Guo B., Lin F., Hui Q., Tao K., Kontos C.K. (2020). Effect of THBS1 on the Biological Function of Hypertrophic Scar Fibroblasts. BioMed Res. Int..

[30] Juárez O.E., Fabiola L.I.C., Leyva-Valencia I., López-Landavery E., García-Esquivel Z., Díaz F., Re-Araujo D., Vadopalas B., Galindo-Sánchez C.E. (2018). Transcriptomic and metabolic response to chronic and acute heat exposure of juvenile geoduck clams *Panopea globosa*. Mar. Genom..

[31] Zoysa M.D., Whang I., Lee Y., Lee S., Lee J.-S., Lee J. (2009). Transcriptional analysis of antioxidant and immune defense genes in disk abalone (*Haliotis discus discus*) during thermal, low salinity and hypoxic stress. Comp. Biochem. Physiol. B.

[32] Vergara-Amadoa J., Silvaa A.X., Manzi C., Nespolo R.F., Cárdenas L. (2017). Differential expression of stress candidate genes for thermal tolerance in the sea urchin *Loxechinus albus*. J. Therm. Biol..

[33] Belin B.J., Lee T., Mullins R.D. (2015). Correction: DNA damage induces nuclear actin filament assembly by Formin -2 and Spire-½ that promotes efficient DNA repair. Elife.

[34] He X., Brakebusch C. (2024). Regulation of Precise DNA Repair by Nuclear Actin Polymerization: A Chance for Improving Gene Therapy?. Cells.

[35] Tobias L., Hiroshi W., Oleg S., Lindgens D., Gee L., Law L., Schmidt H.A., Özbek S., Bode H., Holstein T.W. (2009). Multiple Wnts are involved in Hydra organizer formation and regeneration. Dev. Biol..

[36] Shum C.W.Y., Nong W., So W.L., Li Y., Qu Z., Yip H.Y., Swale T., Ang P.O., Chan K.M., Chan T.F. (2022). Genome of the sea anemone *Exaiptasia pallida* and transcriptome profiles during tentacle regeneration. Front. Cell Dev. Biol..

[37] Al-Shaer L., Leach W., Baban N., Yagodich M., Gibson M.C., Layden M.J. (2023). Environmental and molecular regulation of asexual reproduction in the sea anemone *Nematostella vectensis*. R. Soc. Open Sci..

[38] Trevino M., Harmon S., Burton P. (2011). Wnt Signaling Promotes Oral Fates During Regeneration and Embryogenesis in the Cnidarian *Nematostella vectensis*. Dev. Biol..

