# Supplementary material for: Heat Stress and Anthropogenic Substrates: Molecular and Behavioral Adaptation of Metridium senile in Human-Modified Marine Environments
Source: Int J Mol Sci. 2025 Aug 29;26(17):8415. doi: 10.3390/ijms26178415 (PMC12428564; doi:10.3390/ijms26178415)
Supplement: Supplementary file 1 [file ijms-26-08415-s001.zip › ijms-3768048-supplementary.pdf]

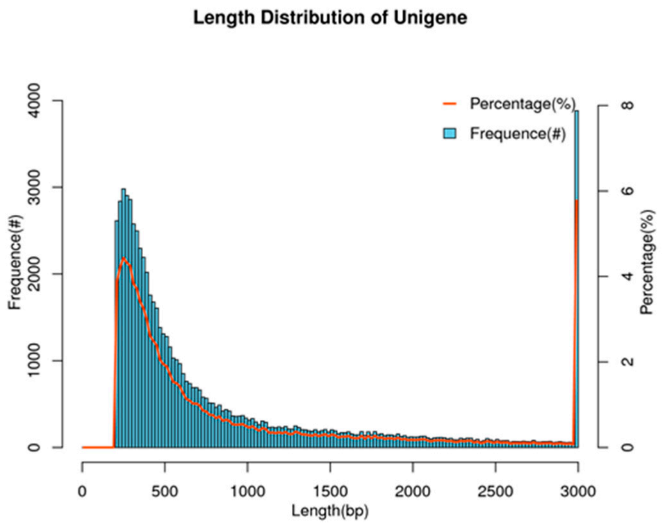

Figure S1 Length distribution of unigenes

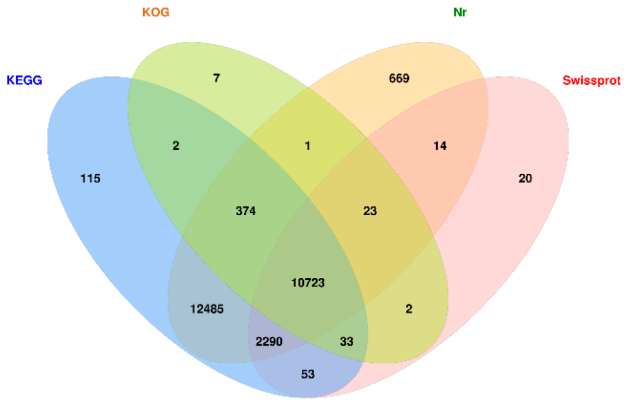

Figure S2 Venn diagram of gene function annotations in different databases

Table S1 Primer information of RT-PCR detection

Primer name	Primer sequence（5’-3’）
18S-F	TCGTATTTCGTTGTCAGAGG
18S-R	TAAGGTGCCAAAGAGGTCAT
TTN-F	GTGGAATGTCCTGCTGCTAG
TTN-R	GTGCGATTAGTTATCGACCC
CTHRC1-F	CCATAGAAGACACGGAGGGC
CTHRC1-R	CTGGTTCGCAAGTCAGTCAA
MMP3-F	CCCCTGATGGCGTTTCCTAT
MMP3-R	CTCCCTTGATGATGAAGATTGC
Col6a6-F	TGTCCCTTGAACCCGATGTG
Col6a6-R	ACCCTGTTTGCGGAAATGTG
TNR-F	AAAGTAGGCTCGTATTCAGG
TNR-R	ATTGGTGCGATACAAGTCAT
Mtnd5-F	ACGGACGAACAGACGCAAAT
Mtnd5-R	TGGTAGTCATGGGTGGAGGC
FMN2-F	TGCGGTTGTGGCTTTATC
FMN2-R	AAGGCGAGGAGGTTGGAC
Csmd3-F	AAATACGATGTAAATCCTGGTG
Csmd3-R	TCATTGCCGTTGAATGCTAG
FCN1-F	CGTACTTTCCGCTGTCCTGG
FCN1-R	GGGCTTCCTGTGCCTTCTGT
Thbs1-F	ACTCACCGCCAAACAACCTC
Thbs1-R	CCGAACAGCTCCTCCACATT

Table S2 Statistics of the clean reads in each experimental group

Temperature	Duplicate	Clean Reads count (Percentage of valid data)	Base number (bp)	GC%	Q30 base percentage
8℃	1	46783368 (99.12%)	6980012516	41.73%	94.18%
	2	41821430 (99.12%)	6241900022	41.57%	93.92%
	3	46887916 (99.41%)	6995891006	41.94%	94.12%
13℃	1	43555328 (99.16%)	6489594570	41.61%	93.81%
	2	38897776 (99.20%)	5791763093	41.88%	93.96%
	3	38369190 (99.17%)	5718459674	41.74%	93.52%
18℃	1	53152090 (99.38%)	7937278839	41.66%	94.55%
	2	51938330 (99.36%)	7749314393	41.60%	94.79%
	3	49406738 (99.34%)	7369700576	41.51%	94.93%

Table S3 Unigenes associated with regeneration

	id	Symbol	Description
1	Unigene0027212	Mtnd5	ABU68799.1 cytochrome b, partial [Tetrahymena thermophila]
2	Unigene0038247	TSPO	XP_032237776.1 translocator protein [Nematostella vectensis]
3	Unigene0046808	ZBTB8OS	XP_020902994.1 protein archease [Exaiptasia diaphana]
4	Unigene0060475	MT-CYB	YP_009243291.1 cytochrome b [Gasterophilus intestinalis]
5	Unigene0063329	FMN2	XP_028514757.1 formin-A-like isoform X1 [Exaiptasia diaphana]
6	Unigene0065270	DDC	XP_028513726.1 aromatic-L-amino-acid decarboxylase [Exaiptasia diaphana]
7	Unigene0073982	--	XP_020900855.1 E3 ubiquitin-protein ligase TRIM71 [Exaiptasia diaphana]

Table S4 Unigenes associated with adhesion

	id	Symbol	Description
1	Unigene0010971	zig-1	XP_020907667.1 neural cell adhesion molecule 1 isoform X1 [Exaiptasia diaphana]
2	Unigene0013288	Ttn	XP_020911695.1 neural cell adhesion molecule 1 [Exaiptasia diaphana]
3	Unigene0015349	HLX	XP_020893172.1 homeobox protein DLX-4 [Exaiptasia diaphana]
4	Unigene0022921	--	KXJ12698.1 Mucin-like protein [Exaiptasia diaphana]
5	Unigene0026552	ADGRD1	XP_028517491.1 adhesion G protein-coupled receptor L1 [Exaiptasia diaphana]
6	Unigene0026690	HMCN1	XP_022783430.1 coadhesin-like [Stylophora pistillata]
7	Unigene0030375	Serpine2	TRY62634.1 hypothetical protein TCAL_10991 [Tigriopus californicus]
8	Unigene0032845	--	KXJ09858.1 Receptor-type tyrosine-protein phosphatase S [Exaiptasia diaphana]
9	Unigene0037894	--	-
10	Unigene0041064	Csmd3	XP_028518080.1 uncharacterized protein LOC110249342 [Exaiptasia diaphana]
11	Unigene0041082	--	XP_020898740.1 histone acetyltransferase KAT6B [Exaiptasia diaphana]
12	Unigene0045947	Thbs1	CAB3266939.1 thrombospondin A precursor [Phallusia mammillata]
13	Unigene0045948	Thbs1	NP_001029015.1 thrombospondin A precursor [Ciona intestinalis]
14	Unigene0051301	ACTB	AAQ05016.1 beta-actin [Tigriopus japonicus]
15	Unigene0054679	--	KXJ12876.1 Fibrillin-2 [Exaiptasia diaphana]
16	Unigene0055657	Atp2a2	XP_019634652.1 PREDICTED: sarcoplasmic/endoplasmic reticulum calcium ATPase 1-like isoform X2 [Branchiostoma belcheri]
17	Unigene0061156	ADAM2	KXJ10236.1 Protein sidekick-2 [Exaiptasia diaphana]
18	Unigene0067726	chrd	XP_028513442.1 chordin [Exaiptasia diaphana]
19	Unigene0069321	TTN	XP_020907667.1 neural cell adhesion molecule 1 isoform X1

			[Exaiptasia diaphana]
20	Unigene0069715	STAB2	XP_028514060.1 stabilin-2 [Exaiptasia diaphana]

Table S5 Unigenes associated with collagen and fibrinogen

	id	Symbol	Description
1	Unigene0001353	CTHRC1	XP_020909418.1 collagen triple helix repeat-containing protein 1 [Exaiptasia diaphana]
2	Unigene0014665	--	XP_031564233.1 cuticle collagen 1-like [Actinia tenebrosa]
3	Unigene0027038	MMP3	XP_020891960.1 72 kDa type IV collagenase [Exaiptasia diaphana]
4	Unigene0030375	Serpine2	TRY62634.1 hypothetical protein TCAL_10991 [Tigriopus californicus]
5	Unigene0030421	FCN1	KXJ25193.1 Fibrinogen C domain-containing protein 1 [Exaiptasia diaphana]
6	Unigene0042141	--	PFX15780.1 Collagen alpha-1(XXVII) chain B [Stylophora pistillata]
7	Unigene0044538	--	KXJ17799.1 Collagen alpha-2(I) chain [Exaiptasia diaphana]
8	Unigene0045948	Thbs1	NP_001029015.1 thrombospondin A precursor [Ciona intestinalis]
9	Unigene0062703	--	XP_020897757.1 collagen triple helix repeat-containing protein 1 [Exaiptasia diaphana]
10	Unigene0067726	chrd	XP_028513442.1 chordin [Exaiptasia diaphana]
11	Unigene0070204	CTHRC1	XP_031559259.1 collagen alpha-3(IV) chain-like [Actinia tenebrosa]
12	Unigene0070507	FIBCD1	XP_028515947.1 uncharacterized protein LOC110242689 [Exaiptasia diaphana]
13	Unigene0072668	Col6a6	XP_028519745.1 collagen alpha-1(XII) chain [Exaiptasia diaphana]
14	Unigene0030421	FCN1	KXJ25193.1 Fibrinogen C domain-containing protein 1 [Exaiptasia diaphana]
15	Unigene0050199	ANGPT2	XP_031573025.1 fibrinogen C domain-containing protein 1-like [Actinia tenebrosa]
16	Unigene0072610	TNR	XP_044178626.1 fibrinogen C domain-containing protein 1-like [Acropora millepora]

Table S6 KEGG enrichment results of DEGs in different temperature groups

	**Pathway**	**Pathway_ID**	**KEGG_A_class**	**KEGG_B_class**
1	ABC transporters	ko02010	Environmental Information Processing	Membrane transport
2	Amino sugar and nucleotide sugar metabolism	ko00520	Metabolism	Carbohydrate metabolism
3	Apoptosis - fly	ko04214	Cellular Processes	Cell growth and death
4	Apoptosis - multiple species	ko04215	Cellular Processes	Cell growth and death
5	Arachidonic acid metabolism	ko00590	Metabolism	Lipid metabolism
6	Arginine and proline metabolism	ko00330	Metabolism	Amino acid metabolism
7	Autophagy - animal	ko04140	Cellular Processes	Transport and catabolism
8	Axon regeneration	ko04361	Organismal Systems	Development and regeneration
9	Base excision repair	ko03410	Genetic Information Processing	Replication and repair
10	Biosynthesis of amino acids	ko01230	Metabolism	Global and overview maps
11	Calcium signaling pathway	ko04020	Environmental Information Processing	Signal transduction
12	Carbon metabolism	ko01200	Metabolism	Global and overview maps
13	Citrate cycle (TCA cycle)	ko00020	Metabolism	Carbohydrate metabolism
14	Dorso-ventral axis formation	ko04320	Organismal Systems	Development and regeneration
15	Drug metabolism - cytochrome P450	ko00982	Metabolism	Xenobiotics biodegradation and metabolism
16	Drug metabolism - other enzymes	ko00983	Metabolism	Xenobiotics biodegradation and metabolism
17	ECM-receptor interaction	ko04512	Environmental Information	Signaling molecules and

			Processing	interaction
18	Endocytosis	ko04144	Cellular Processes	Transport and catabolism
19	Ether lipid metabolism	ko00565	Metabolism	Lipid metabolism
20	Folate biosynthesis	ko00790	Metabolism	Metabolism of cofactors and vitamins
21	FoxO signaling pathway	ko04068	Environmental Information Processing	Signal transduction
22	Glutathione metabolism	ko00480	Metabolism	Metabolism of other amino acids
23	Glycerolipid metabolism	ko00561	Metabolism	Lipid metabolism
24	Glycerophospholipid metabolism	ko00564	Metabolism	Lipid metabolism
25	Glycine, serine and threonine metabolism	ko00260	Metabolism	Amino acid metabolism
26	Glycolysis / Gluconeogenesis	ko00010	Metabolism	Carbohydrate metabolism
27	Hippo signaling pathway - fly	ko04391	Environmental Information Processing	Signal transduction
28	Linoleic acid metabolism	ko00591	Metabolism	Lipid metabolism
29	Longevity regulating pathway - worm	ko04212	Organismal Systems	Aging
30	Lysine biosynthesis	ko00300	Metabolism	Amino acid metabolism
31	Lysine degradation	ko00310	Metabolism	Amino acid metabolism
32	Lysosome	ko04142	Cellular Processes	Transport and catabolism
33	MAPK signaling pathway	ko04010	Environmental Information Processing	Signal transduction
34	Metabolic pathways	ko01100	Metabolism	Global and overview maps
35	Metabolism of xenobiotics by cytochrome P450	ko00980	Metabolism	Xenobiotics biodegradation and metabolism
36	Neuroactive ligand-receptor	ko04080	Environmental Information	Signaling molecules and

	interaction		Processing	interaction
37	Nicotinate and nicotinamide metabolism	ko00760	Metabolism	Metabolism of cofactors and vitamins
38	Nitrogen metabolism	ko00910	Metabolism	Energy metabolism
39	Non-homologous end-joining	ko03450	Genetic Information Processing	Replication and repair
40	Notch signaling pathway	ko04330	Environmental Information Processing	Signal transduction
41	Nucleocytoplasmic transport	ko03013	Genetic Information Processing	Translation
42	One carbon pool by folate	ko00670	Metabolism	Metabolism of cofactors and vitamins
43	Oxidative phosphorylation	ko00190	Metabolism	Energy metabolism
44	Peroxisome	ko04146	Cellular Processes	Transport and catabolism
45	Phagosome	ko04145	Cellular Processes	Transport and catabolism
46	Phenylalanine metabolism	ko00360	Metabolism	Amino acid metabolism
47	Phototransduction - fly	ko04745	Organismal Systems	Sensory system
48	Purine metabolism	ko00230	Metabolism	Nucleotide metabolism
49	Pyrimidine metabolism	ko00240	Metabolism	Nucleotide metabolism
50	Pyruvate metabolism	ko00620	Metabolism	Carbohydrate metabolism
51	Retinol metabolism	ko00830	Metabolism	Metabolism of cofactors and vitamins
52	Spliceosome	ko03040	Genetic Information Processing	Transcription
53	TGF-beta signaling pathway	ko04350	Environmental Information Processing	Signal transduction
54	Tryptophan metabolism	ko00380	Metabolism	Amino acid metabolism
55	Tyrosine metabolism	ko00350	Metabolism	Amino acid metabolism
56	Valine, leucine and isoleucine	ko00290	Metabolism	Amino acid metabolism

	biosynthesis			
57	Wnt signaling pathway	ko04310	Environmental Information Processing	Signal transduction
58	alpha-Linolenic acid metabolism	ko00592	Metabolism	Lipid metabolism
59	mRNA surveillance pathway	ko03015	Genetic Information Processing	Translation
60	mTOR signaling pathway	ko04150	Environmental Information Processing	Signal transduction
